# A multi-omics approach to unravel the interaction between heat and drought stress in the *Arabidopsis thaliana* holobiont

**DOI:** 10.3389/fpls.2024.1484251

**Published:** 2024-12-19

**Authors:** Biancamaria Senizza, Fabrizio Araniti, Simon Lewin, Sonja Wende, Steffen Kolb, Luigi Lucini

**Affiliations:** ^1^ Department for Sustainable Food Process, Università Cattolica del Sacro Cuore, Piacenza, Italy; ^2^ Dipartimento di Scienze Agrarie e Ambientali, Produzione, Territorio, Agroenergia (Di.S.A.A.), Università degli Studi di Milano, Milano, Italy; ^3^ Microbial Biogeochemistry, Research Area Landscape Functioning, Leibniz Center for Agricultural Landscape Research – Leibniz Center for Agricultural Landscape Research e.V. (ZALF), Muencheberg, Germany; ^4^ Thaer Institute, Faculty of Life Sciences, Humboldt University of Berlin, Berlin, Germany

**Keywords:** heat stress, drought, multi-omics, phytohormones, Proteobacteria, *Rhizobiales*

## Abstract

The impact of combined heat and drought stress was investigated in *Arabidopsis thaliana* and compared to individual stresses to reveal additive effects and interactions. A combination of plant metabolomics and root and rhizosphere bacterial metabarcoding were used to unravel effects at the plant holobiont level. Hierarchical cluster analysis of metabolomics signatures pointed out two main clusters, one including heat and combined heat and drought, and the second cluster that included the control and drought treatments. Overall, phenylpropanoids and nitrogen-containing compounds, hormones and amino acids showed the highest discriminant potential. A decrease in alpha-diversity of Bacteria was observed upon stress, with stress-dependent differences in bacterial microbiota composition. The shift in beta-diversity highlighted the pivotal enrichment of *Proteobacteria*, including *Rhizobiales*, *Enterobacteriales* and *Azospirillales*. The results corroborate the concept of stress interaction, where the combined heat and drought stress is not the mere combination of the single stresses. Intriguingly, multi-omics interpretations evidenced a good correlation between root metabolomics and root bacterial microbiota, indicating an orchestrated modulation of the whole holobiont.

## Introduction

Heat and drought are two environmental stresses that occur with climate change-associated extreme weather events, which substantially impact plant growth and development (Giordano et al., 2021). Drought stress occurs due to an imbalance between the evapotranspiration flux and water intake, mainly when the soil water availability and the atmospheric humidity are low and the air temperature is high. Hence, both stresses often occur simultaneously. The plant response to drought stress depends on the species, plant growth stage, and environmental factors (Fahad et al., 2017). Heat stress, defined as the rise in soil and air temperature beyond a threshold level for a minimum amount of time (Lamaoui et al., 2018), may inactivate enzymes and cause damage to proteins and changes in their synthesis. Moreover, heat stress could have major effects on cell division processing. Most experimental studies have focused on a single stressor due to the challenging biological cross-talk between the multiple plant responses and their interpretations.

Plants are always colonized by complex microorganism communities in their root system (i.e. the plant microbiota) that, together with their plant host, build the holobiont (Lyu et al., 2021). It is well known that the rhizosphere is colonized by a broad diversity of microorganisms which may accumulate depending on the stress level of the holobiont (Francioli et al., 2021). Bacteria are one dominant group of rhizomicrobiota, known to directly interact with the host plant by various mechanisms impacting growth and the plant immune system (Rizaludin et al., 2021). Indeed, through roots, plants exude a mixture of small molecules that select specific portions of soil bacteria (Zhang et al., 2021). Such a “Cry for help strategy” suggests that stressors lead to changed signaling and substrate release in the root system and rhizosphere to acquire beneficial microbes (Rizaludin et al., 2021). This active recruitment of microorganisms improves resilience to abiotic stresses by eliciting physiological, biochemical, and molecular responses in the plant’s local and distal parts (Meena et al., 2017) (Saeed et al., 2021). Noteworthy, single and combined abiotic stresses may indirectly alter plant functions via the modulation of plant- and root-associated microbiota (Rahman et al., 2021), triggering substantial changes in plant development (Francioli et al., 2021; Lewin et al., 2021). Despite plant adaptation processes and the microbiota responses to abiotic stresses being known, the effect of combined stressors on holobionts is still poorly investigated (Rivero et al., 2022).

Plant responses to such stresses are complex and may not correspond simply to the sum of the two abiotic stresses applied individually (Zandalinas et al., 2020), but they can interact and negatively impact plants even if the effect of each stress is slight (Zandalinas et al., 2021). For instance, the response to two different stresses applied simultaneously to two different leaves of the same plant was different and more extensive than the response to two different stresses applied individually (Balfagón et al., 2019). Furthermore, increased atmospheric CO_2_ concentrations reduce the impact of combined heat and drought stress on *Arabidopsis* spp., activating antioxidant defense mechanisms and reducing photorespiration (Zinta et al., 2018). These complex and interconnected responses involve several molecular and physiological modifications and acclimation to include systemic signaling, accumulation of stress-specific transcripts, and hormones. The gene expression of heat shock proteins (HSPs) (Georgii et al., 2017) is altered and occurs in different ways when stresses are combined (Zandalinas et al., 2017).

Hence, our work aimed at investigating the impact of heat, drought, and their combination on the plant holobiont, considering plant-specific metabolites and hormones, as well as the root and rhizosphere bacterial microbiota. Recently, a holo-omics approach has been suggested to assess simultaneously in one experimental design both the plant host and its microbiota response to environmental changes to better understand changed interactions and the relevance of enriched microbial taxa for its host plant (Xu et al., 2021). Accordingly, a holo-omic approach was applied as an integrated analytical strategy to resolve the coordinated and complex dynamic interactions between the plant and its rhizosphere bacteria, using *Arabidopsis thaliana* as a model plant species.

## Materials and methods

### Plant growth and morpho-physiological assays

The experiments were carried out on *Arabidopsis thaliana* plants (cv Columbia 0) following the protocol proposed by Rizhsky et al. (2004) with some modifications. A professional potting soil (Orticole alveolo TecnoGrow, Tercomposti, Calvisano BS, Italy) was sterilized before the experiment (120°C for 20 min). Before sowing, seed germination was synchronized by soaking the seeds in sterile water for 76 hours at 4°C in dark conditions. Ten seeds were then sown per pot, and after germination, they were thinned, leaving one seedling per pot. A total of 24 pots (5 x 5 x 5 cm) were prepared for each treatment and replicate.

Seedlings were grown in a growth chamber under controlled conditions: 12 *h* light/12 h dark photoperiod (long day), 21 ± 1°C, 100 µmol m^-2^ s^-1^, and relative humidity of 60-70%. Seedlings were fertilized every other day through sub-irrigation using a half-strength Hoagland solution and grown for 22 days *Arabidopsis thaliana* growth stage 3.50, Rosette is ~ 50% final size (Boyes et al., 2001).

The following treatments were applied: C (Control watered plants), D (Drought stress), H (heat stress), and D+H (Drought + Heat stress). Drought treatment was achieved by blocking plant irrigation until they reached a relative water content (RWC) of 65% to 70% (typically 5–6 d). In contrast, heat stress was applied by raising the temperature gradually (~ 4°C per hour) to avoid heat shock, in the growth chamber to 35°C, and then keeping the plants at this temperature for 12 h.

At the end of the experiments, 1 g of the rhizosphere and root samples were collected for the microbiome analysis. Roots were carefully washed in sterilized distilled water and immediately snap-frozen in liquid nitrogen. Both soil and powdered roots were stored at -80°C (Thijs et al., 2017).

During the experiments, morphological and physiological parameters were monitored. In particular, the changes in biomass were evaluated at the end of the experiments on the fully developed rosette, measuring the changes in fresh weight (FW), dry weight (DW) and the DW/FW ratio. Another indicator of plant stress was the chlorophyll content, carried out at the end of the experiment using a chlorophyll meter SPAD (MC100, Qingdao Tlead International Co. Ltd). Finally, to have an indicator of plant water status and the plant’s ability to preserve it, the temperature of the leaves was monitored using a thermocamera (FLIR), and the leaf relative water content (% RWC) as previously reported by Araniti et al., 2020.

### Untargeted metabolomics analysis

*Arabidopsis thaliana* roots were extracted using a homogenizer-assisted extraction in 80% methanol solution with 0.1% (v/v) formic acid, centrifuged and filtered through 0.22 µm cellulose filters. The phytochemical profile of roots was investigated through ultra-high-pressure liquid chromatography (UHPLC) coupled with quadrupole-time-of-flight (QTOF) mass spectrometry, as previously reported by Senizza et al., 2021. Briefly, the mobile phase consisted of a mixture of water and acetonitrile (both LC-MS grade, VWR, Milan, Italy) acidified with 0.1% (v/v) formic acid, with a gradient from 6 to 94% of acetonitrile in 35 min. An injection volume of 6 μl and a pentafluorophenylpropyl column (2.0 × 100 mm, 3 µm - Agilent Technologies, Santa Clara, CA, USA) were used. The mass spectrometer acquired ions in the range 100-1200 m/z in positive scan mode (ESI+) at a rate of 0.8 spectra/s (40,000 FWHM, absolute peak height threshold 5000 counts).

For raw data processing, the software Profinder B.07 (Agilent Technologies) was used, considering monoisotopic mass (25-ppm tolerance for mass accuracy), isotope spacing and ratio according to the “find-by-formula” algorithm. Mass and retention time alignment, as well as compound filtering, were performed before compounds annotation. The database PlantCyc was used as a reference for annotations (Hawkins et al., 2021). Only the compounds identified in 100% of replications within at least one condition were retained in the dataset and used further. According to COSMOS Metabolomics Standards Initiative, the annotation process corresponded to a Level 2 of identification (i.e., putatively annotated compounds) (Salek et al., 2015).

### The rhizosphere microbiota structure by metabarcoding

The DNeasy PowerLyzer PowerSoil Kit (Qiagen, Hilden, Germany) was used to extract DNA from roots and soil samples. The amplification of bacterial DNA was carried out by LGC genomics GmbH (Berlin, Germany) using the primers 799f and 1115r, and the amplicons were sequenced on an Illumina MiSeq instrument with 300bp paired-end reads.

Demultiplexing was conducted with Illumina bcl2fastq 2.17.1.14 software following the clipping of barcode and sequencing adapters. Primers were removed using Cutadapt v3.0 (Martin, 2011). Sequences were processed in R 4.1 with dada2 version 1.22.0 (Callahan et al., 2016). Due to adapter ligation-based library prep, the raw sequences were in mixed orientation. To get the correct final orientation for learning error rates, reads were split into two groups (forwardRead.forwardPrimer - reverseRead.reversePrimer, and reverseRead.forwardPrimer - forwardRead.reversePrimer), denoised separately and merged after chimera removal. Forward and reverse reads were truncated at positions 265 and 210, resulting in 4073 unique Amplicon sequencing variants (ASV). Taxonomic classification was performed using the q2-feature-classifier plugin from Qiime2 version 2021.8.0 with a naïve Bayes classifier trained on the Silva 138.1 NR99 database.

### Statistical analysis

All the experiments were carried out in a completely randomized design with 5 replications. The univariate analysis of morphological and physiological parameters was carried out using XLSTAT 2014.5.03. Data were analyzed through one-way ANOVA using Duncan’s test as *post hoc* (P ≤ 0.05).

Concerning metabolomics, the post-acquisition data analysis was carried out using the software Mass Profiler Professional 12.6 (Agilent Technologies); the compounds were log2 transformed, normalized at the 75th percentile, and baselined against the median. Afterwards, both unsupervised and supervised multivariate statistics were applied for interpretations. According to Euclidean distance and Ward’s linkage, the unsupervised hierarchical cluster analysis was used to underline the relatedness across the different treatments. In addition, the supervised orthogonal projection to latent structures discriminant analysis (OPLS-DA) was carried out, and the model parameter (goodness-of-fit R^2^Y and goodness-of-prediction Q^2^Y) were recorded. Also, the OPLS-DA model was cross-validated (CV-ANOVA), inspected for outliers (Hotelling’s T2), and the overfitting was excluded through a permutation test (n = 200). Then, the Variable Importance in Projection (VIP) analysis was used to select the metabolites having the highest discriminant potential (VIP score > 1.2). Finally, the differential compounds obtained from the ANOVA and fold-change analysis (FC) (p < 0.05, Bonferroni multiple testing correction and Fold-Change FC ≥ 2) were exported into the Omic Viewer Pathway Tool of PlantCyc (Plant Metabolic Network) (Stanford, CA, USA) software for interpretation (Karp et al., 2009; Caspi et al., 2013).

Regarding metabarcoding data, downstream analysis was performed using RStudio with R version 4.1.1. Phyloseq v1.38.0 was used to handle ASV sequences and tables. Samples were split into compartments (soil, root) and separately analyzed. After outlier removal (one sample from control treatment on root and in soil, one sample from drought treatment and two from Drought-Heat treatment due to small sampling size), ASVs were filtered for mitochondria, and unassigned sequences were removed. Only ASV that (a) occurred in at least 3 samples and (b) occurred >10 times in total were retained for downstream analysis, leading to 1455 ASV in soil samples and 915 ASV in root samples. Alpha diversity indices (number of observed ASVs, Inverse Simpson index) were calculated using rarefied and filtered samples from root and soil datasets and plotted by treatment. Bray-Curtis dissimilarity indices were calculated on rarefied relative abundances and used to perform principal coordinate analysis (PCoA) and permutational analysis of variance (PERMANOVA) to investigate treatments’ effect on the bacterial community structure. For PERMANOVA, 999 permutations were carried out per dataset. Linear discriminant analysis of effect size (LEfSe) was applied to the root and soil datasets aggregated to the Genus level to identify keystone taxa that drive the differences between treatments (Segata et al., 2011). LEfSe was run with a Wilcoxon and Kruskal-Wallis cut-off value of 0.01. An LDA cut-off value of 2 resulted in 182 and 145 marker genera for root and soil, respectively, and an LDA cut-off of 4 in 43 and 10 marker genera. In addition, analysis of compositions of microbiota with bias correction (ANCOMBC) was used to identify differentially abundant features (Lin and das Peddada, 2020). The Holm-Bonferroni method was applied to adjust p-values, and features with adjusted *P* values <0.01 were considered significant, resulting in 193 and 313 genus-level markers for root and soil datasets, respectively.

### Combined discriminant analysis of metabarcoding and metabolomics datasets

Data Integration Analysis for Biomarker discovery using Latent variable approaches for Omics studies (DIABLO) from the package “mixOmics” was used for the integration of metabolomics and root metabarcoding datasets (Rohart et al., 2017). This supervised approach allowed the integrated analysis of multiple datasets and was used to identify discriminant features in both datasets that drive differences between treatment groups. Values of the design matrix were set to 0.1 to prioritize the discriminant ability of the model. Center log ratio (clr) transformation was applied to both datasets, and root metabarcoding data was aggregated at the genus level beforehand. An optimal number of 3 components for “centroid.dist” distance was determined using the function perf() with 6-fold cross-validation and 10 repeats. The number of features selected for sparse PLS-DA was tuned with the function tune.block.splsda() using 4-fold cross-validation with 10 repeats. The features selected for each component were 18, 40, 6 for metabarcoding and 6, 14, 90 for metabolomics. The correlation among components of each dataset was checked with plotArrow().

## Results

### Morpho-physiological parameters

**Figure 1:**
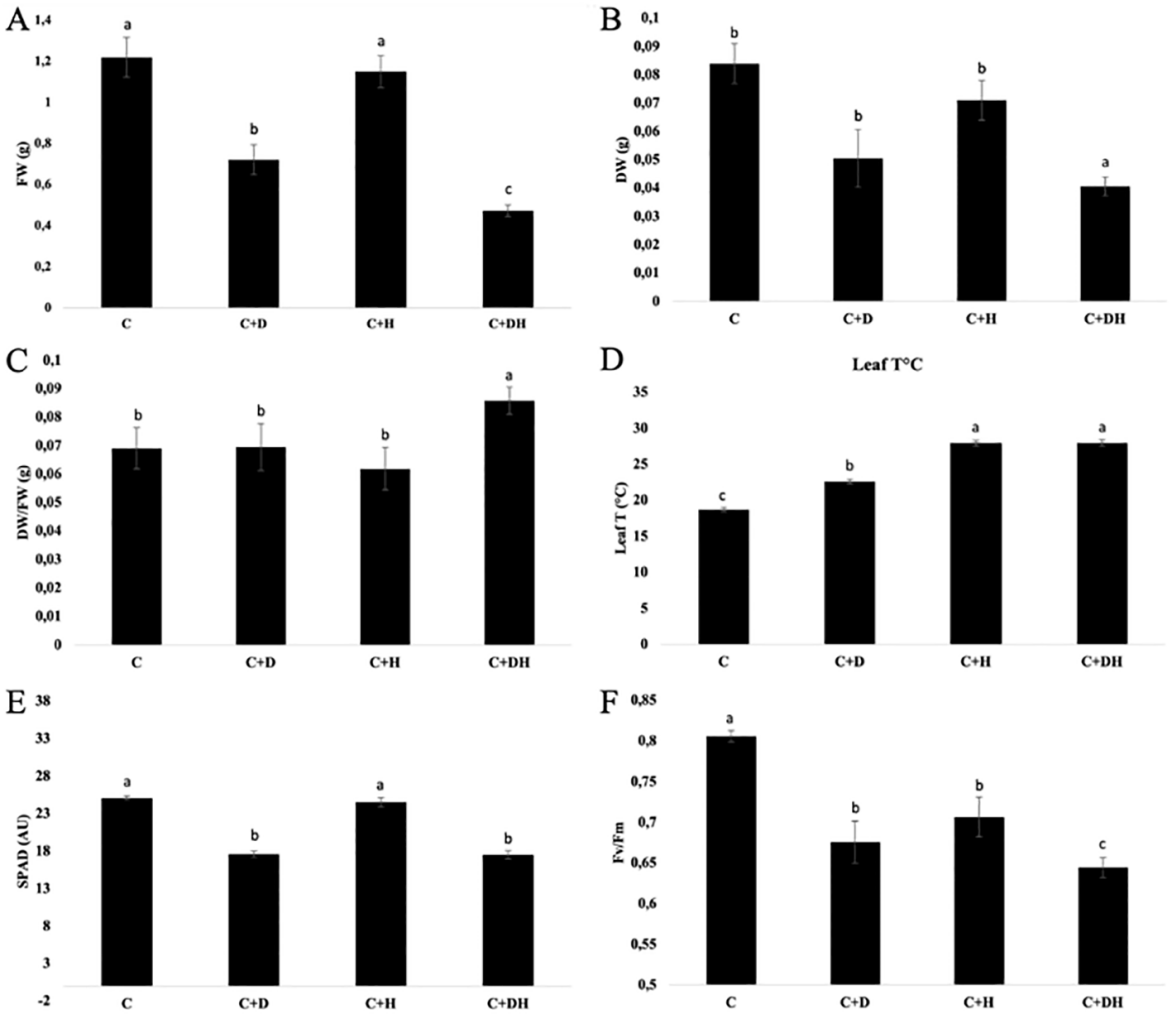
Effects of single and combined stress on *Arabidopsis thaliana* on shoot freshweight (FW) **(A)**, shoot dry weight (DW) **(B)**, FW/DW **(C)**, leaf temperatur **(D)**, SPAD **(E)** and Fv/Fm **(F)**. C (watered plants); C+D (C + drought stress); C+H (C + heat stress); C+H+D (stress combination). Data were analyzed through one way ANOVA using the LSD’s test as *post-hoc*. Different letters along the bars indicate statistical differences with P ≤ 0.05. N=4.

The experiment lasted until the loss of turgidity in the leaves of the stressed control. Firstly, the data underlined a significant decrease of the FW in plants under D, halved compared to C, and in the combined stress (C+H+D), being about three times lower than the control (C) (**Figure 1A**). Regarding the DW and the FW/DW ratio, only C+H+D was significantly different (p<0.05) from the other treated plants (**Figures 1B, C**).

In addition, the leaves temperature increases in all the stressed plants, in particular the increase was higher in C+H and C+D+H (**Figure 1D**). The chlorophyll content measurement was performed randomly measuring SPAD on 5 leaves per plot. The SPAD index was significantly (p<0.05) lower in drought-stressed plants and in C+H+D, with a decrease of ∼30% compared to the watered control (C) (**Figure 1E**). Considering the C+H, the chlorophyll content was not significantly different (P<0.05). Finally, the Fv/Fm ratio decreased for stressed plants, mostly in the combined stress (C+H+D) with a reduction of ∼25% (**Figure 1F**).

### Untargeted metabolomics profiling of *Arabidopsis thaliana* roots by UHPLC/QTOF-mass spectrometry

The untargeted metabolomics approach was used to investigate the effect of heat and drought stresses and their combination on the metabolomic profile of *A. thaliana* roots. The metabolomics raw data are submitted to a public repository (https://www.ebi.ac.uk/metabolights/MTBLS6421). This approach allowed us to putatively annotate more than 1100 compounds, further used to infer the biological processes involved in plant stress responses. The list of the annotated metabolites, with composite mass spectra and abundance, is provided as supplementary material (**Supplementary Table S1**).

**Figure 2:**
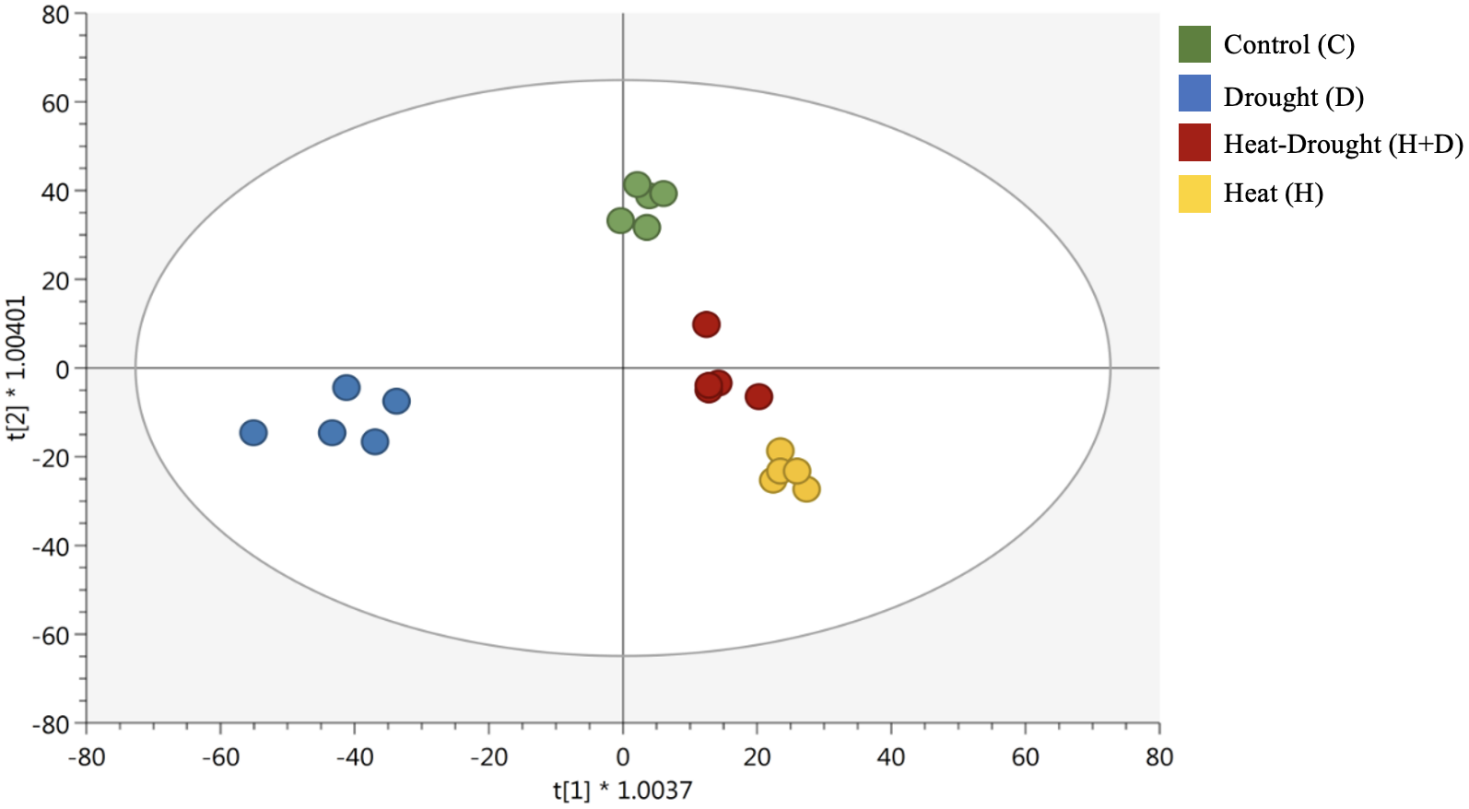
Score plot of orthogonal projection to latent structures discriminant analysis (OPLS-DA) supervised modeling carried out on untargeted metabolomics profiles of *Arabidopsis thaliana* roots exposed to heat, drought, and combined stresses (R2Y = 0.96, Q2Y = 0.91). The symbol * indicates a the symbol for multiplication.

Unsupervised hierarchical cluster analysis and supervised OPLS discriminant analysis were successfully performed to identify patterns between the conditions under investigation. The clusters produced from the heatmap based on fold-change highlighted distinct metabolomic profiles in roots, depending on the stress applied. In fact, two main clusters were generated, one featuring H and H+D and the other including the control and D (**Supplementary Figure S1**). These findings were corroborated by the supervised OPLS-DA, where all stress conditions separated from the control and underlined a separation of D from the other conditions (control, H and H+D) (**Figure 2**).

The score plot also showed that the combined stress (H+D) influenced metabolic profile closer to the H and far from the control and D. Afterwards, VIP analysis was carried out to find the compounds with the highest contribution to the OPLS-DA discrimination (VIP score > 1.2, **Supplementary Table S2**). Overall, phenylpropanoids and nitrogen-containing compounds, hormones, and amino acids exhibited the highest discriminant potential.

**Figure 3:**
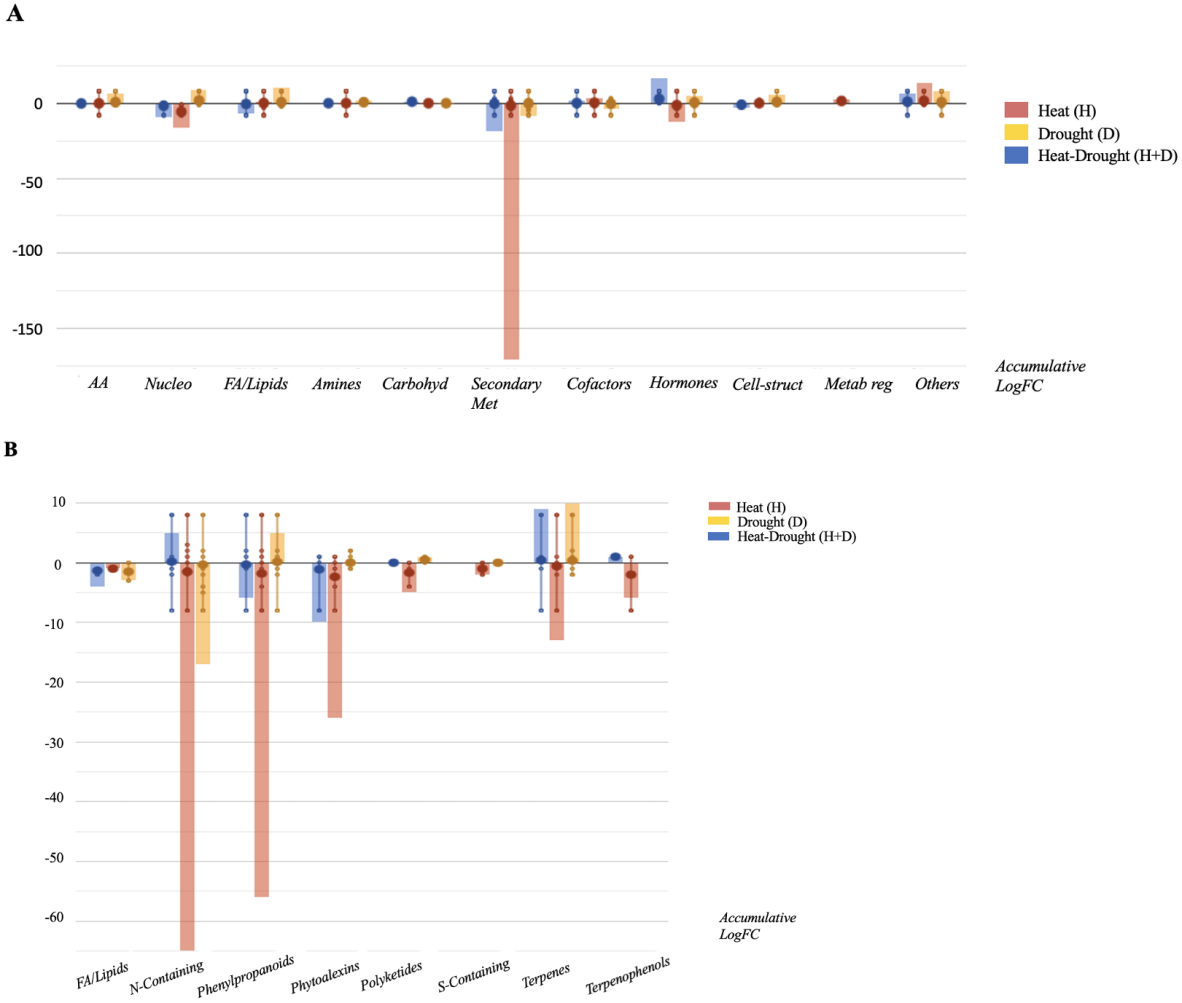
Metabolic processes **(A)** and specialized metabolite biosynthesis **(B)** modulated *Arabidopsis thaliana* roots exposed to heat, drought and the combined stresses (heat+ drought). The metabolomics dataset produced through UHPLC-ESI/QTOF-MS was subjected to ANOVA and FC analysis (p < 0.05, FC ≥ 2), and differential metabolites were loaded into the PlantCyc Pathway Tool (https://www.plantcyc.org/).

Then, Volcano Plot analysis identified 405 differential compounds significantly differing from the control (p-value < 0.05; FC ≥ 2). Despite the large number of compounds modulated in response to the stresses, only 65 metabolites overlap for all the H, D and H+D (**Supplementary Table S3**). Moreover, the combined treatment (H+D) presented several compounds common with H stress, indicating a hierarchical prevalence of the latter. In contrast, D alone provoked a distinct response, as suggested by OPLS-DA. **Figure 3** depicts the modulation of the biosynthetic pathways in response to the specific stresses resulting from differential compounds accumulation. Overall, all the individual and combined stress had a detrimental effect on root metabolism, particularly specialized metabolism. In agreement with multivariate analysis, roots exposed to H stress showed a comparatively stronger modulation of specialized metabolism. In general terms, several plant defense mechanisms were activated in a stress-specific manner. Despite the general decrease of specialized metabolites, glucosinolates increased in response to stress (H, D, H+D). In this sense, phytoalexins related to glucosinolates pathways (i.e. indole-3-carboxaldehyde) and glutathione-related compounds were also modulated under combined stress conditions (H+D).

Phenylpropanoids decreased in response to H and, to a lesser extent, to H+D while increasing in response to D, which promoted the accumulation of flavonoids and anthocyanins. Regarding terpenes, H decreased these compounds while H+D and D alone elicited this pathway, including their precursors. Although the detrimental effect of H on plant metabolism, plants exposed to H increased in phospholipids while sterols accumulated in the two other stress conditions considered (D, H+D).

**Figure 4:**
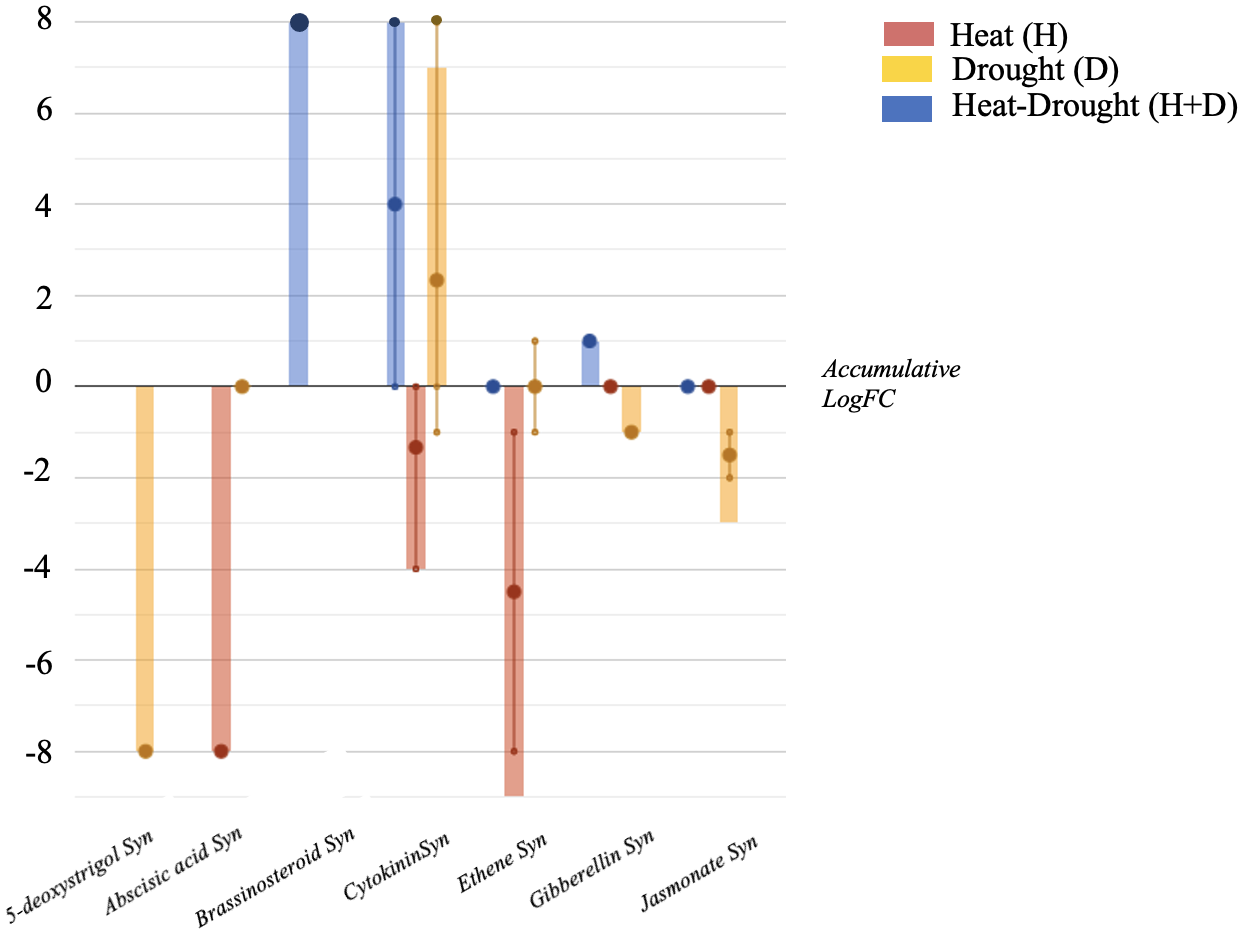
Hormones biosynthesis modulated in *Arabidopsis thaliana* roots exposed to heat, drought and the combined stresses (heat + drought). The metabolomics dataset produced through UHPLC-ESI/QTOF-MS was subjected to ANOVA and FC analysis (p < 0.05, FC ≥ 2), and differential metabolites were loaded into the PlantCyc Pathway Tool (https://www.plantcyc.org/). The x axis represents each set of metabolic subcategories, while the y axis corresponds to the accumulative log fold change (FC). The large dots represent the average (mean) of all FCs for the different metabolites in the class, while the small dots represent the individual log FC.

The stresses also influenced the phytohormone profile, with distinctive modulations as a function of the condition considered (**Figure 4**). In more detail, cytokinins (CKs) were accumulated by the combined stress (H+D) and D stress. Moreover, in H, CKs and abscisic acid (ABA) synthesis were negatively modulated, while gibberellins (GAs) and jasmonates (JAs) were down accumulated under water deficit conditions (D). Ascorbate-related compounds were also modulated in plants exposed to the stress, with dehydroascorbate accumulated under H and D stress.

### Bacterial richness and biodiversity

In soil and root samples, 1455 and 915 bacterial ASVs remained after filtering out sparse ASVs and outliers, respectively. These AVSs were affiliated with 20 phyla, 39 classes, 87 orders, 130 families and 184 genera in root and 27 phyla, 51 classes, 114 orders, 170 Families and 238 Genera in soil.

**Figure 5:**
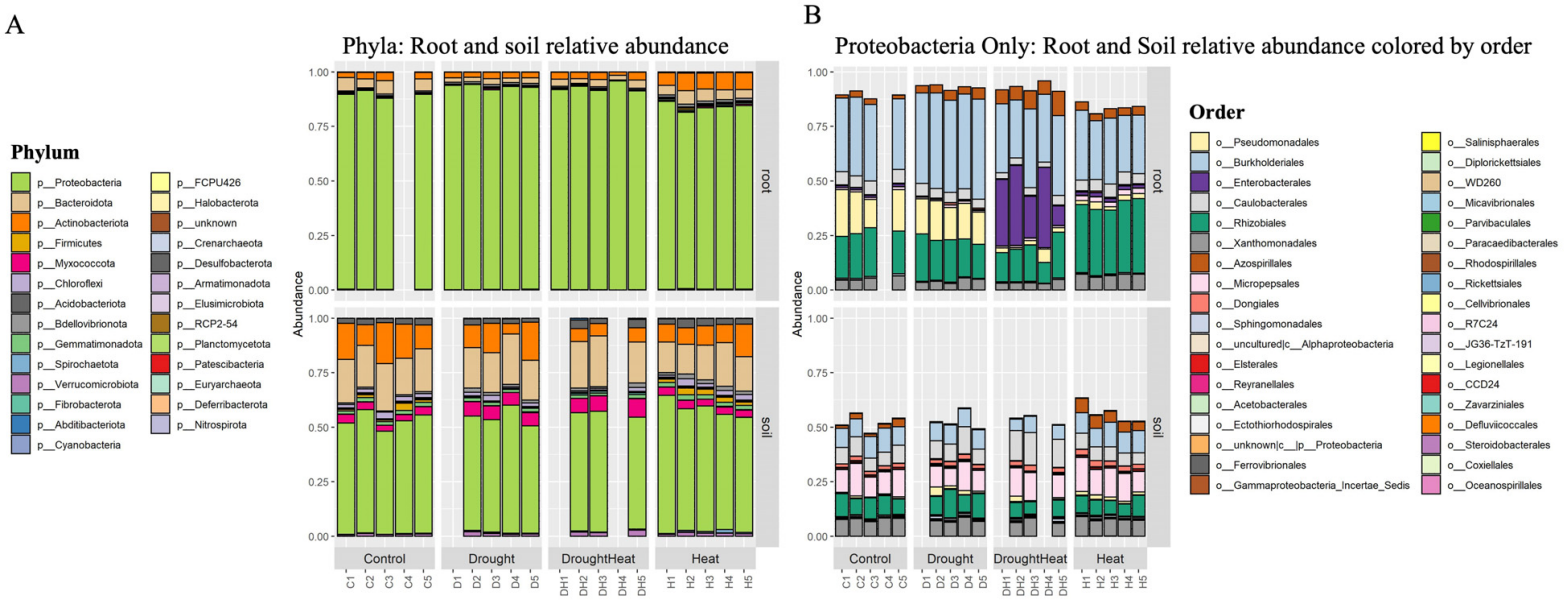
Taxa barplots for samples from root and rhizosphere soil. Relative abundances of bacterial taxa identified based on reference to SILVA database v138. Prior to plotting abundance tables were filtered for rare ASVs and outlier samples were removed. **(A)** Phylum level barplot shows relative abundances of identified taxa aggregated on phylum level for root (top) and soil (bottom) compartments for each replicate grouped by treatment. The bacterial microbiota was dominated by Proteobacteria. **(B)** Relative abundances of only Proteobacteria, colored by order level for a detailed resolution of Proteobacteria taxa composition.

Both soil and root samples were dominated by *Proteobacteria*, which made up, on average, 89% of classified reads in root samples and 53% in soil samples (**Figure 5A**). Taking a more detailed look at the distribution of orders within the Proteobacteria (**Figure 5B**), pronounced different patterns between the compartments (root, soil) as well as the treatments (C, H, H+D, D). In soil samples, a large fraction of *Micropepsales* (average of 11.5% of reads) was detected, which is substantially less frequent in root samples (1.2% of reads). In the roots, treatment differences also refer to proteobacteria, mainly classified as *Rhizobiales* and *Burkolderiales*. A large fraction of *Pseudomonadales* occurs in both the C and D treatments. The combined H+D treatment only was characterized by a substantial increase in Enterobacteriales (26.4% of reads), which were also present in the heat treatment to a lesser extent (mean 1.56%).

**Figure 6:**
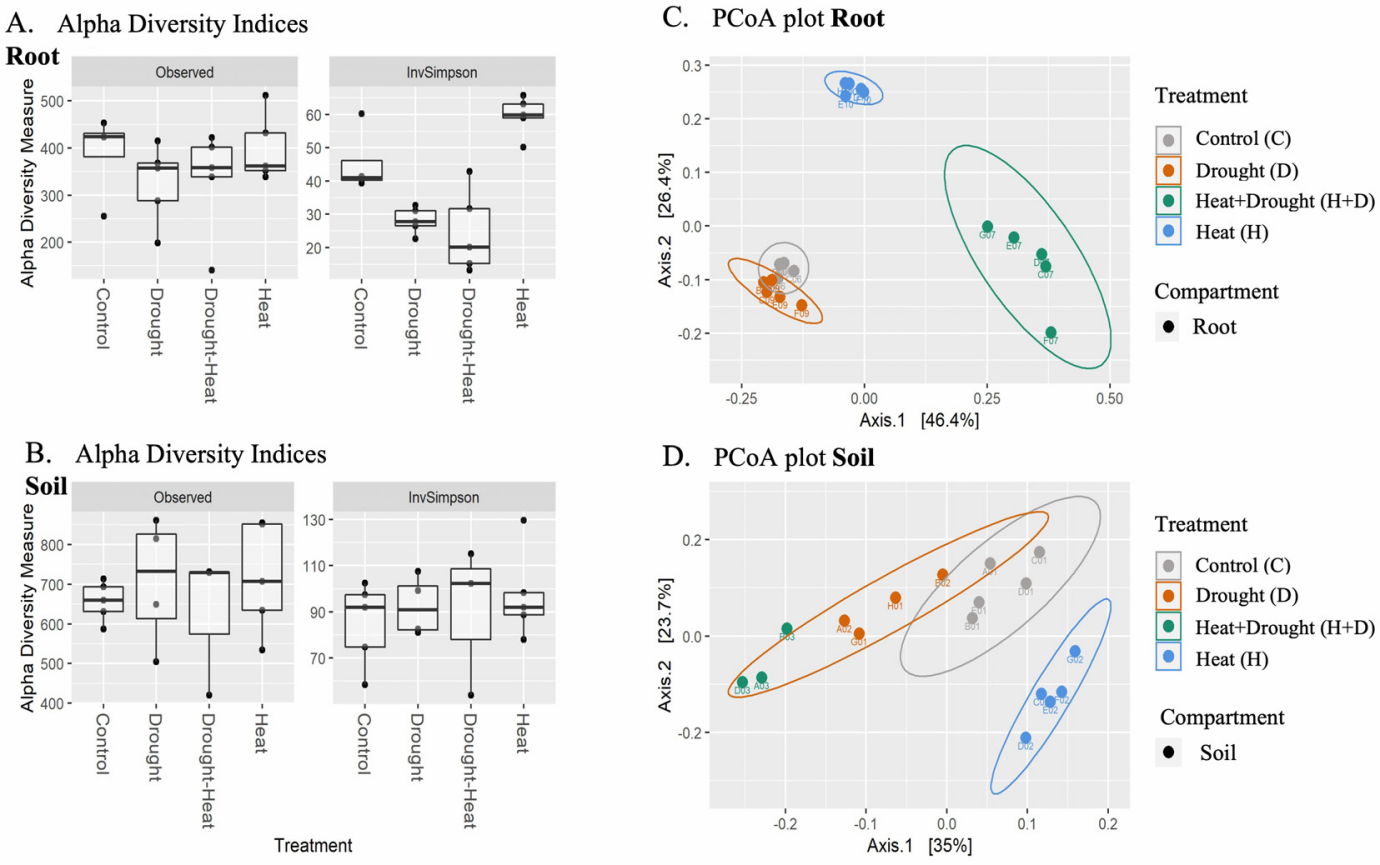
Boxplots of alpha and beta diversities for treatment groups based on observed ASV and Inverse Simpson index for **(A)** root and **(B)** soil. Alpha diversity indices were calculated based on filtered, rarefied data. Bray Curtis dissimilarity indices were calculated for filtered and rarefied root **(C)** and soil **(D)** datasets and plotted using PCoA. Samples groups have a 95% confidence ellipse drawn around them.

A decrease in bacterial alpha-diversity was observed from the rhizosphere to the root (**Figures 6A, C**). Considering the different stress, differences in alpha-diversity were evident under drought stress (with lower values than the not stressed control). Analysis of variance (ANOVA) followed by Tukey’s honestly significant difference (HSD) *post hoc* test revealed no effect of the treatments on the richness in either compartment. However, a significant interaction between treatment and inverse Simpson index for the root samples was identified (p≥0.05).

The beta-diversity was determined using principal coordinate analysis (PCoA) with Bray-Curtis dissimilarities (**Figures 6B, D**). The first two axes accounted for over 72% and 58% of the variance between treatments of root and soil samples, respectively. PERMANOVA analysis on the root and soil datasets indicated a significant effect of all treatments, where drought alone explains 23.2% of the variance, heat 38.7% and the combination 22.2% in root samples. In soil samples, D explains 29.1% of the variance, heat only 18.9% and combined H+D only 13.6% of the variance (p≥0.001) (**Supplementary Table S4**). Then, linear discriminant analysis effect size (LEfSe) was carried out to identify the taxa driving the differences between treatments (**Supplementary Figure S2**) and the ANCOM-BC differential analysis was used to identify the taxa differentially abundant between the treatments. Both methods perform a similar analysis but look at the problem differently. As expected, the marker taxa established as significant were largely shared between the methods, being 131 in roots and 138 in soil (**Supplementary Figure S3**). For interpretation, we focused then on LefSE results to identify keystone taxa that explain differences between sample groups.

In root samples, LefSe analysis revealed 21 microbial biomarkers in C, 11 in D, 71 in H and 14 in H+D.The most discriminant taxa in H belonged to the phyla of *Actinobacteriota* and *Proteobacteria*, in particular, *Rhizobiales*, *Xanthomonadales* and *Streptomycetales*, while *Burkholderiales* characterized the D samples. The combined stress (H+D) was characterized by the presence of *Gammaproteobacteria*, being the *Enterobacterales and Azospirillaleles* the most discriminating taxa. However, non-stressed roots (C) were characterized by *Pseudomanadales* and *Caulobacterales*. (**Supplementary Figure S4A**).

In soil samples there were 12 microbial biomarkers detected in C, 9 in D, 20 in H and 25 in H+D. Here the most discriminant taxa for H were the alphaproteobacterium *Azospirillum* and the phylum *Firmicutes* member of *Bacillales* and *Clostridiales*. At the same time, H+D was most defined by a taxon from *Polyangiales*, followed by *Rhizobiales, Fibrobacterales, Sphingobacteriales* and *Chitinophagales*, and member from the phylum *Elusimicrobiota.* In D differences were driven by *Devosiaceae* and *Cryseobacterium* and C by *Geobacterales* and *Ktedonobacterales* (**Supplementary Figure S4B**).

### MultiOmics integration

**Figure 7:**
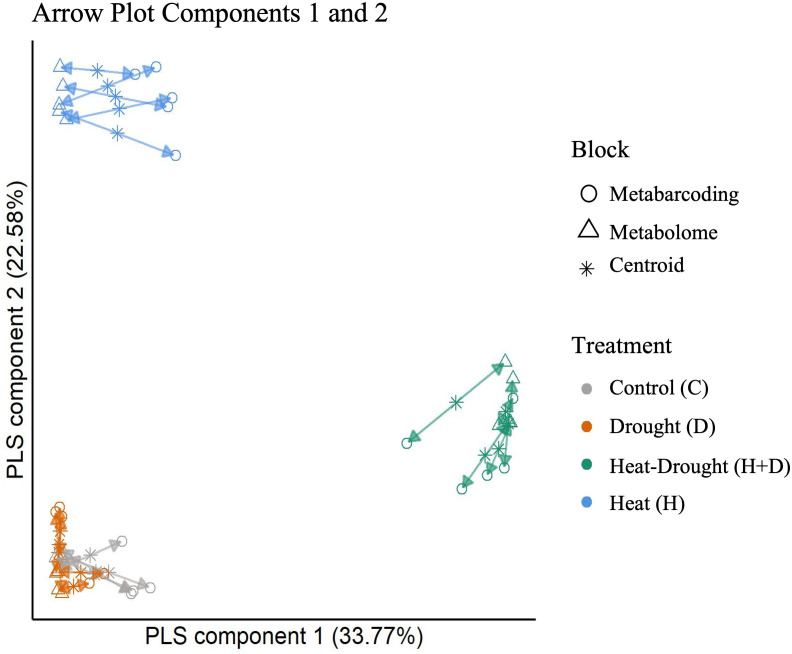
Arrow Plot from multiblock sPLS-DA (DIABLO). Samples of the data blocks metabarcoding and metabolomics are plotted into the space spanned by the first two components of the model. The length of the arrows indicates the distance of each sample from the centroids of both datasets. Short distances indicate high levels of agreement between data metabolomics and metabarcoding blocks.

Following the holo-omics approach, we integrated the metabolomics and root metabarcoding datasets using the DIABLO framework (Singh et al., 2019) with multiblock sparse PLS-DA (partial least squares discriminant analysis). Model tuning helped us to select features from metabarcoding and metabolomics to improve the modelling of differences between groups. Noteworthy, the datasets are highly correlated for all three components (**Figure 7**, components 1 and 2, **Supplementary Figure S5**, components 2 and 3). The agreement between metabarcoding and metabolomics is high for all samples and treatments. The combined drought and heat treatment separates from the other three treatments on the first axis, and heat treatment on the second axis, while the drought and control treatments are not well separated by components 1 and 2, but are separated by component 3 (**Supplementary Figure S5**).

As a follow-up analysis to DIABLO, Pearson correlation network analysis was performed. We aimed to identify positive correlations between features assigned to the same treatment by DIABLO. These pairs likely resolve the underlying interaction between the plant metabolome and the rhizosphere microbiota.

Only bacterial genera and plant metabolites significantly assigned to a feature by DIABLO were included in network construction. Three clustering methods (optimal, Louvain, fast greedy clustering) reproduced four similar modules. The largest was mainly constituted by correlations between features of the control treatment and several heat or drought metabolites. The latter were likely false positives of the DIABLO analyses.

Besides the control module, only metabolites and genera assigned as features of the H+D treatment by DIABLO co-occurred in a single module. Thus, crosslinks between metabolite and bacterial microbiota datasets were characteristic of the H+D treatment (**Figure 7**).

Additionally, a fraction of drought-associated bacterial genera and heat-associated metabolites formed connections within this majorly H+D-associated module. The module consisted of 24 nodes in total (**Table 1**). Among them were predominantly nitrogen-containing secondary compounds for biosynthesis, phenylpropanoid derivative biosynthesis, polyketide biosynthesis and *Proteobacteria*.

**Table 1:** Nodes network module H+D.

Node	Genus/Metabolite	Phylum/Pathway
CPD-12423	2-sinapoyloxy-3-butenylglucosinolate	Nitrogen-Containing Compounds Biosynthesis
CPDQT-406	(E)-1-(L-cysteine-S-yl)-N-hydroxy-omega (methylsulfanyl)pentan-1-imine	Nitrogen-Containing Compounds Biosynthesis
GenASV17	*Azospirillum*	p_Proteobacteria
GenASV128	*Noviherbaspirillum*	p_Proteobacteria
GenASV58	*Delftia*	p_Proteobacteria
GenASV100	o_Enterobacterales|f_|g_un	p_Proteobacteria
GenASV5	f_Enterobacteriaceae|g_un	p_Proteobacteria
GenASV176	*Pseudonocardia*	p_Actinobacteriota
CPD-11566	heptaketide pyrone	Polyketide Biosynthesis

**Figure 8:**
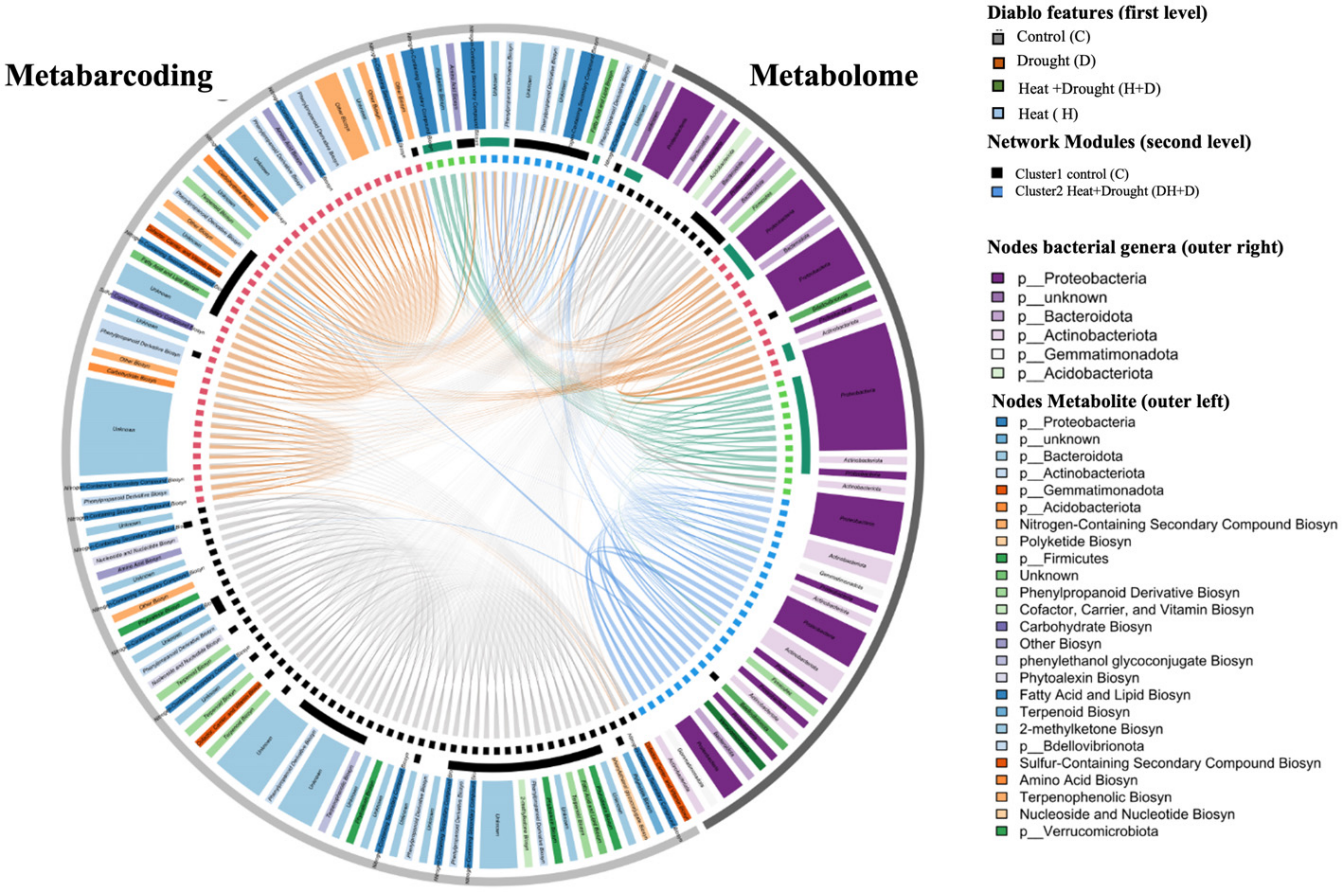
Circular heatmap shows median abundance values of selected features within treatment groups drought (D), Heat (H), Drought-Heat (H+D) and control (C). Color track inside shows the feature phylum association for metabarcoding block and functional categories for metabolome features. Chord diagram depicting pearson network analyses of plant metabolites and bacterial genera identified as significant features during DIABLO analyses for treatments. Chords represent correlation larger than 0.7 with size scaled to r. Inner circle shows network nodes colored b DIABLO features, second level indicates network modules representative of C and H+D treatment. Outer circle describes phylum and class of Bacteria and metabolites.

In contrast, the majority of drought-associated metabolites or heat-associated bacterial genera formed no crosslinks between the same feature category across the metabolite and metabarcoding datasets. Instead, drought-associated metabolites correlated with other metabolites and heat-associated bacterial genera interacted with other bacteria, as indicated by characteristic arcs within **Figure 8**.

In conclusion, a small core of *Proteobacteria* was associated with nitrogen-containing secondary compounds in response to combined H+D stress. The combined treatment appears to further select plant metabolism responsive to heat metabolism, but rhizosphere bacteria responsive to D.

## Discussion

Plants are sessile organisms responding to environmental stimuli and stresses with consequent rearrangement of the metabolism and an impact on the root-associated and rhizosphere microbiota. These two aspects are tightly connected and can be considered two perspectives of the same response since specific plant metabolites like phenolics, glucosinolates, and strigolactones can shape soil microbes and their interactions (Hartman et al., 2017; Jacoby et al., 2021).

A distinctive adjustment of the specialized metabolism was observed depending on the stress, with more pronounced effects in roots subjected to thermal stress (H). Under limited water conditions (D), oxidative stress may induce the synthesis of phenolic compounds and flavonoids via the increase in the activity of phenylalanine ammonia-lyase (PAL), the key upstream enzyme of the phenylpropanoids pathway (Jun et al., 2018). Despite showing a general down accumulation of phenolics during thermal stress, our results indicate some sub-classes being enhanced, thus suggesting a specific modulation of this class of metabolites. Nonetheless, accumulating other low molecular weight antioxidants such as ascorbate and glutathione may help the plant mitigate ROS-mediated damage related to limited transpiration related to drought. Overall, changes in phenolic acids and flavonoids also depend on the species, the intensity of the stress and its duration. Recent work demonstrated that different wheat genotypes under water scarcity did not exhibit any significant change in phenolic acids and flavonoids, while others had higher concentrations than non-stressed ones (Laddomada et al., 2021). In addition, a higher phenolic acids content was detected under severe drought conditions, while moderate drought and severe heat stress did not lead to their accumulation (Shamloo et al., 2017).

A general down accumulation was observed in both the single stress conditions concerning N-containing compounds. However, glucosinolates and related compounds were accumulated. These compounds have been reported to potentially affect the rhizosphere communities (Jacoby et al., 2021). Interestingly, the production of aliphatic glucosinolates is induced by drought in *Arabidopsis thaliana*, in parallel with phytoalexins repression (Kliebenstein, 2004). Together with glucosinolates, a slight modulation of amino acids synthetic pathways was observed in response to single stresses. Under abiotic stress, amino acids may accumulate as precursors for synthesizing specialized metabolites and signaling molecules or as substrates for protein synthesis to promote rapid plant metabolism recovery from stress. Among them, L-methionine was reported to be an effective regulator of plant growth under environmental stress such as drought (Mehak et al., 2021).

Several phytohormones such as jasmonic acid (JA), abscisic acid (ABA), brassinosteroids (BRs), cytokinins (CKs), and gibberellic acid (GA) have been shown to enhance abiotic stress tolerance (Sharma et al., 2019). In fact, under high temperature and/or water deprivation conditions, plant responses are mediated by phytohormones, which coordinate complex stress-adaptive signaling cascades (Vile et al., 2012) through a complex cross-talk between the different signaling pathways (Verma et al., 2016). Several studies focused on hormonal changes during a combination of high temperatures and salinity or high light intensity, agreeing that a coordinated hormonal response to each specific stress combination is essential to trigger the proper acclimation responses. In our conditions, ABA was involved in the plant response to heat and drought stress. Zandalinas et al., 2016 revealed that although ABA is required for the acclimation of Arabidopsis, stomatal closure may also be regulated by H_2_O_2_. A cross-talk mechanism between gibberellin and abscisic acid during limited water conditions was also reported, in which ABA biosynthesis and the control of stomatal conductance were regulated by the receptor for gibberellin under water stress (Gaion et al., 2018). These latter are involved in the adaptive response to various abiotic stresses such as cold, salinity, heat, flooding, and drought, despite their role in drought stress adaptation is still unclear. A cross-talk between ABA and BRs has also been reported (Ha et al., 2016). However, decreasing CKs (negative regulators of plant root growth and branching) under H stress can improve plant survival rate by reducing the expression of stress response genes (Liu et al., 2020). Similarly, also jasmonates have a significant role in abiotic stress tolerance because of their linkage with other growth regulators, antioxidants and osmoprotectants, especially its conjugate isoleucine-JA, the most active form of JAs (Sharma et al., 2019). Notably, phytohormones may also be related to microbial colonization and play a pivotal role in the assembly of plant microbiota (Eichmann et al., 2021). In turn, the ability of beneficial microorganisms to directly produce CKs, GAs, and ABA, rather than aminocyclopropane-1-carboxylase (ACC) deaminase that cleaves the ethylene precursor, can support plant growth under stress (Pascale et al., 2020; Vishwakarma et al., 2020; Eichmann et al., 2021).

Plants’ acclimation to a particular abiotic stress condition involves tailored responses to their specific environmental conditions. Previous literature indicates that a moderate overlap was observed among transcriptomic responses to abiotic stressors such as drought (Rizhsky et al., 2004), cold (Kreps et al., 2002), salinity (Yong et al., 2002) and light excess (Rossel et al., 2002). Similarly, despite being recognized as a common response to abiotic stressors, the shift in gene expression patterns related to ROS-triggered responses was differently modulated by stress treatments (Mittler et al., 2004). As in our case, several studies have shown that combined stress responses are mostly related to non-additive effects. Our findings revealed that H and D stress largely modulated root metabolism and triggered different plant responses, while the combined stress did not imply a sum of both stresses but presented a metabolic profile closer to those plants exposed to heat stress. These findings agree with previous studies and confirm that the combination of abiotic stresses is rarely the sum of the single stresses (Fahad et al., 2017; Lamaoui et al., 2018; Zandalinas et al., 2018; Laddomada et al., 2021). Indeed, the review by Mittler on abiotic stress combination reports how unique stresses cannot be used to extrapolate combined stresses (Mittler et al., 2004). To date, little information is available on the molecular mechanisms underlying the interaction between abiotic stresses and plants response, even though the comprehension of such mechanisms is important to facilitate the development of crops with enhanced stress tolerance (Rizhsky et al., 2004).

Regarding the specific stress combination in our experiments, the interaction between drought and high temperature is realistic in the context of a climate change scenario, and it is also relevant in terms of stress interaction. During heat stress, stomata are opened to increase transpiration and cool leaves; however, the combination with drought hampers this heat dissipation mechanism and represents a clear example of positive interaction. Surprisingly, our results indicate the hierarchically prevalent effect of heat while confirming the positive interaction. Consistently with our findings on multivariate modelling of the plant metabolome, the interaction between heat and drought is believed to provide unique responses (Rizhsky et al., 2002) and should be considered an independent stress condition. Indeed, tolerance to combined stresses involves the cross-talk among different signal transduction processes that requires multiple controls. This latter point, reflecting synergistic relationships among stresses, has been defined as “cross-hardening” (Bowler and Fluhr, 2000). As highlighted by our data, the integration between stresses is rather complex; it involves signaling processes like hormones, mitogen-activated protein kinases (MAPK), and calcium and ROS species (Bowler and Fluhr, 2000). Moreover, plants employ complex “stress sensing” mechanisms to detect stresses, depending on the species, organ, and type of stress (Kranner et al., 2010) and the cross-talk between receptors may also be involved (Casal, 2002).

Together with highlighting the non-additive effect of multiple stresses, our results strengthen the concept of whole holobiont response to stress where a coordinated plant-microbiome modulation was observed. Accordingly, the combined effect of heat and drought stress on the rhizosphere microbiome produced a different outcome compared to single stressors. Concerning rhizobacteria, the literature on their role in plant abiotic stress mitigation is vast, as recently reviewed (Navarro et al., 2006; Dimkpa et al., 2009). At the molecular level, plant perception of eubacterial flagellins can activate plant responses at the gene expression level. Xu et al., suggested that at the earlier developmental stage, the roots bacterial microbiota is more susceptible than at older plant stages (Xu et al., 2018), although mitigation features are stress-dependent and not a *per se* feature of the strains (Rolli et al., 2015). While heat affects the rhizosphere microbiota via the host plant (indirectly), drought shapes the bacterial microbiota, directly promoting the enrichment of Bacteria belonging to *Firmicutes* and *Actinobacteria* (Simmons et al., 2020), which are known to be physiologically adapted to drought conditions and that their abundance is positively correlated to plant drought resistance (Naylor et al., 2017; Fitzpatrick et al., 2018; Hartman and Tringe, 2019). Moreover, in accordance with our results, it has been recently reported that under drought stress, endophytic *Actinobacteria* induced artemisinin biosynthesis, which accumulation is known to be involved in modulating drought tolerance (Li et al., 2012).

Plants actively exudate compounds that act as attractants for *Rhizobium* species and that may be used as carbon sources by other species, including *Burkholderia*, and their breakdown products might modify the microbial biodiversity and the species abundance (Schütz et al., 2021). These rhizhobiales-plants interactions significantly mitigate abiotic stresses (Munir et al., 2022). Our results on beta diversity highlighted the involvement of *Proteobacteria*, known to be the main members of *Arabidopsis’* root microbiota both in the roots and in the soil, followed by *Bacteoroidetes* and *Actinobacteria*, as a function of the stress applied. As expected, the magnitude of bacterial microbiota shifts was consistently lower in soil than in root-associated compartments.

The involvement of the root endophyte *Enterobacteriales*, stimulated by both H and H+D treatments, in mediating plant thermotolerance has been recently described (Shekhawat et al., 2021). They reported that *Enterobacter* sp. SA187 enhanced the H3K4me3 levels at heat stress memory gene loci, which was mediated by ethylene signaling. Similarly, Proteobacteria such as *Aeromonas* sp., which use flavonoids-mediated signaling for host recognition, have been proven to be an enhancer of plant dehydration resistance (He et al., 2022).

*Actinobacteria* possess a drought-tolerant nature and, under stress, increase the transcription of specific genes and the production of spores highly tolerant to dehydration (Omae and Tsuda, 2022). Consistently, we observed an increase in *Actinobacteria*, especially under H treatment. Among monoderm lineages, *Actinobacteria* exhibit the strongest enrichment under abiotic stress and support plant carbohydrate and amino acid transport and metabolism, as well as to positively modulate plant secondary metabolism (Ngumbi and Kloepper, 2016; Xu et al., 2018). The mechanisms by which *Actinobacteria* mitigate abiotic stress in plants include the production of osmolytes to maintain osmotic balance, the synthesis of plant hormones, and enhanced availability of nutrients (Fitzpatrick et al., 2018).

*Proteobacteria*, given their high ability to utilize root exudates, are fast-growing rhizosphere and root colonizers that respond rapidly to organic carbon sources (Bulgarelli et al., 2013). Despite having a relatively superior colonization ability within the root and rhizosphere under well-watered conditions, diderm bacteria are less suited to survive the selective pressures caused by drought (Xu et al., 2018). However, significant differences are present at the genus level, where the structure of the peptidoglycan cell wall, rather than the presence or absence of an outer membrane, can determine significant differences among microorganisms (Sutcliffe, 2010; Xu et al., 2018). Interestingly, the thickness and composition of the cell wall have been linked to a different tolerance to ROS species, one of the mechanisms proposed in the differences observed at the rhizomicrobiota level under abiotic stress (Shade et al., 2012; Fitzpatrick et al., 2018). Consistently, proteobacteria were extensively altered by the different stress conditions, including the genera *Pseudomonas* (decreasing), *Azospirillium*, *Rhizobium*, *Enterobacter*, and *Burkholderia* (all increasing in both roots and soil). Beneficial Bacteria like *Azospirillales*, *Rhizobiales* and *Burkholderia* (all involved in beta-diversity shifts following H and H+D) also led to the production of osmoprotectants like proline, betaine, trehalose, and glycine (Zulfiqar et al., 2020). Similarly, *Enterobacter* can promote stress tolerance, likely because of its phosphate-solubilizing ability (Dolkar et al., 2018). In our experiment, *Enterobacterales* were predominant in H+D stressed roots, while *Rhizobiales* characterized H conditions. At the molecular level, these microorganisms can increase plant biomass under abiotic stress by shaping the phytohormone profile and improving the antioxidant machinery (Brilli et al., 2019).

Despite the evident coordinated modulation of plant metabolome and metabolome in root-associated compartments, our results must be considered in the framework of a strong niche differentiation during plant development. Indeed, the root and rhizosphere bacterial microbiota undergo an initial period of dynamic recruitment followed by a later period of relative stability. The roots grown under pre-flowering drought treatment showed a more pronounced reshape of the microbiota than post-flowering drought treatments (Xu et al., 2018; Francioli et al., 2021; Lewin et al., 2021; Francioli et al., 2022). While confirming the orchestrated response of the whole holobiont to a combination of abiotic stressors, based on the studies mentioned above and our work, it would be very promising to investigate dynamics occurring as a function of plant stage at root and rhizosphere microbiota level.

## Conclusions

Plants adapt to unfavorable environmental conditions by dynamic development at physiological, biochemical, and morphological levels. These complex responses involve several signaling compounds, metabolites, transcription factors, and hormones. Our results strengthen the concept of stress interaction, pointing out as the combined heat and drought stress is not merely the combination of the single stresses but rather the result of a multi-level interaction that involves specialized metabolites and a complex remodeling of phytohormones. Intriguingly, the metabolomics signatures were shaped in a coordinated manner with the root and rhizosphere bacterial microbiota structural shifts, which suggested that the complete plant holobiont should be considered when studying the responses of plants to abiotic stresses. Given the untargeted nature of our metabolome analyses and the subsequent annotation of compounds (i.e., not identification), further research is advisable using additionally targeted approaches to further strengthen our findings. Our study eventually suggest that a complex series of stress sensors and signaling processes likely orchestrate the responses at both plant and microbiota level to optimize plant microbiota assembly and thus, to facilitate a harmonized modulation of stress mitigation processes.

## Data Availability

The names of the repository/repositories and accession number(s) can be found below: NCBI BioProject PRJNA900448, accession numbers SRR22261982 to SRR22262021, as well as https://www.ebi.ac.uk/metabolights/MTBLS7365.
