# Supplementary material for: A multi-omics approach to unravel the interaction between heat and drought stress in the Arabidopsis thaliana holobiont
Source: Front Plant Sci. 2024 Dec 19;15:1484251. doi: 10.3389/fpls.2024.1484251 (PMC11693709; doi:10.3389/fpls.2024.1484251)
Supplement: Supplementary file 5 [file DataSheet1.pdf]

Supplementary Material

A multi-omics approach to unravel the interaction between heat and drought stress in the *Arabidopsis thaliana* holobiont

Biancamaria Senizza[1], Fabrizio Araniti[2], Simon Lewin[3], Sonja Wende[3], Steffen Kolb[3,4*], Luigi Lucini[1]

*** Correspondence:** Steffen Kolb: Steffen.Kolb@zalf.de

1 Supplementary Tables

Supplementary Table S1: Whole dataset produced from untargeted metabolomics carried out in *Arabidopsis* roots exposed to heat, drought and the combined stress. Compounds are listed with individual intensities and with composite mass spectra (monoisotopic accurate mass/abundance combinations).

Supplementary Table S2: Discriminant metabolites (VIP markers) identified by the OPLS-DA analysis in Arabidopsis roots exposed to heat, drought and combined stress.

Supplementary Table S3: Differential metabolites derived from ANOVA and fold-change (FC) analysis (p-value < 0.05, Benjamini multiple testing correction; fold-change threshold FC ≥ 2) in roots metabolomic profiles after stress exposure. These compounds were uploaded into the Omic Viewer Pathway Tool of PlantCyc (Plant Metabolic Network, http://www.plantcyc.org/.

Supplementary Table S4: Bray-Curtis dissimilarity indices were calculated on rarefied relative abundances and used to perform principal coordinate analysis (PCoA) and permutational analysis of variance (PERMANOVA) to investigate stress effect on the bacterial community structure

2 Supplementary Figures

Supplementary Figure S1: Unsupervised hierarchical cluster analysis (Euclidean distance; linkage rule: Ward) carried out from root chemical profiles exposed to heat, drought and the combined stress. Metabolites were obtained by UHPLC-ESI/QTOF-MS untargeted analysis, and their intensities used to build up the fold-change heatmap here provided.

Supplementary Figure S2: Cladogram of LEfSe Biomarker analysis in root. Taxonomic nodes that explain the most variance and are indicative for a certain treatment are colored by treatment. Each ring represents a taxonomic level starting from phylum (p__) over class (c__), order (o__), family (f__) to genus (g__). The described analysis was performed with LDA score cut-off of 3, but only marker with an LDA score > 4 are depicted in this cladogram to avoid an overcrowded image.

Supplementary Figure S3: Venn diagram showing the number of features shared between LefSe (red) and ANCOMBC (grey) analyses. Filtered soil and root datasets were aggregated at genus level and analysed by discriminant analysis (LEfSe, grey) with LDA cut-off set to 2. For differential analysis p-

values were adjusted for multiple testing using holm-Bonferroni method and p-value cutoff of 0.01 was chosen the define significance.

Supplementary Figure S4: Plotted effect Size of Biomarkers identified by LefSE analysis for A) root and B) soil with an LDA score over 3. Color indicates the treatment they discriminant for size of dot represent p-value. The marker taxonomic lineage is displayed on 3 levels.

Supplementary Figure S5: Multiblock sPLS-DA (DIABLO). Samples from data blocks metabarcoding and metabolomics are plotted into space spanned by the second and third components. Length of the arrows indicate the distance of each sample from the centroids of both datasets. Short distances show a high level of agreement between blocks. For all samples and treatments, the agreement between metabarcoding and metabolomics is high.
