# Supplementary figures and images for: A multi-omics approach to unravel the interaction between heat and drought stress in the Arabidopsis thaliana holobiont
Source: Front Plant Sci. 2024 Dec 19;15:1484251. doi: 10.3389/fpls.2024.1484251 (PMC11693709; doi:10.3389/fpls.2024.1484251)

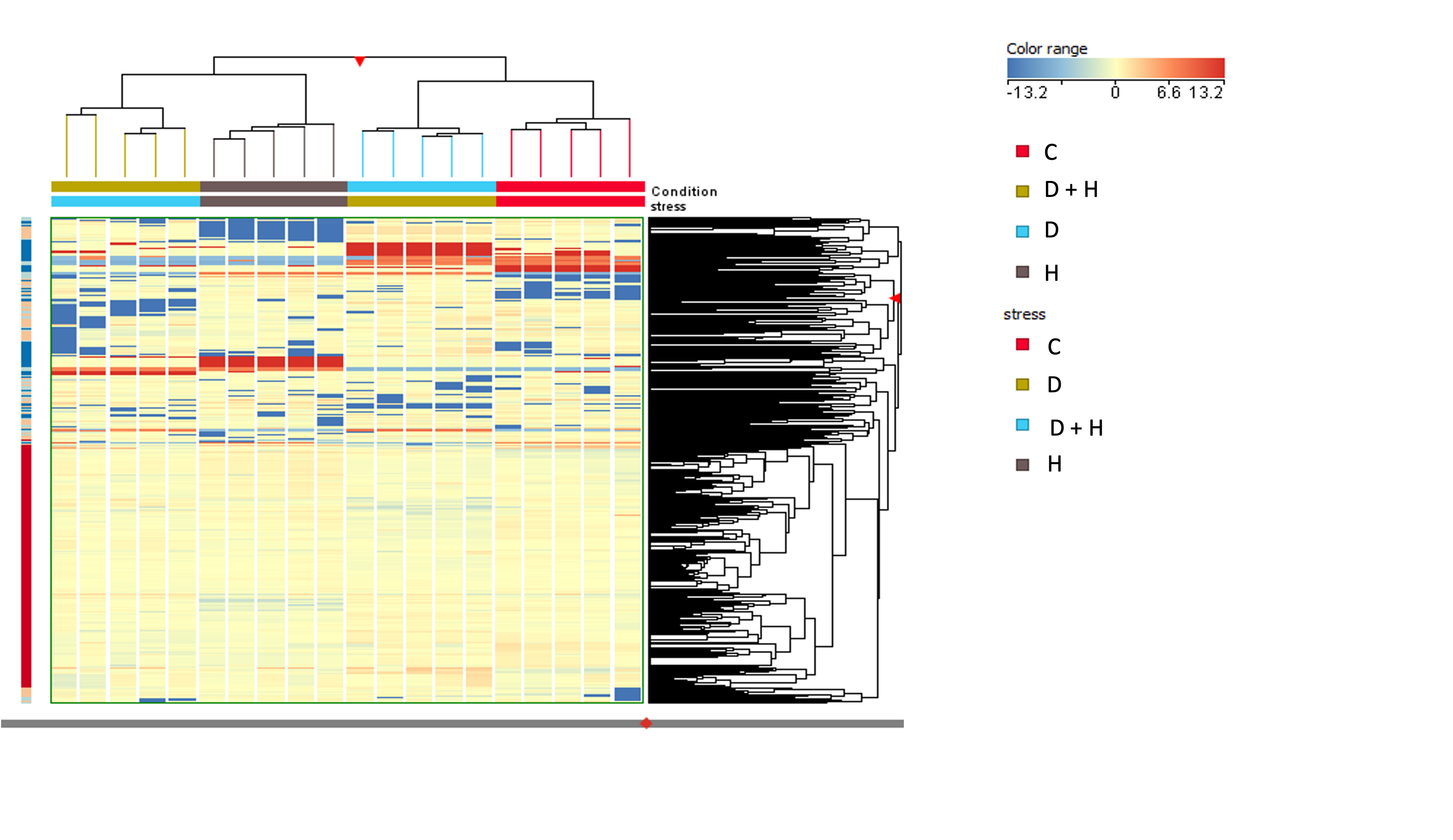

Supplement: Supplementary file 6 [file Image1.png]

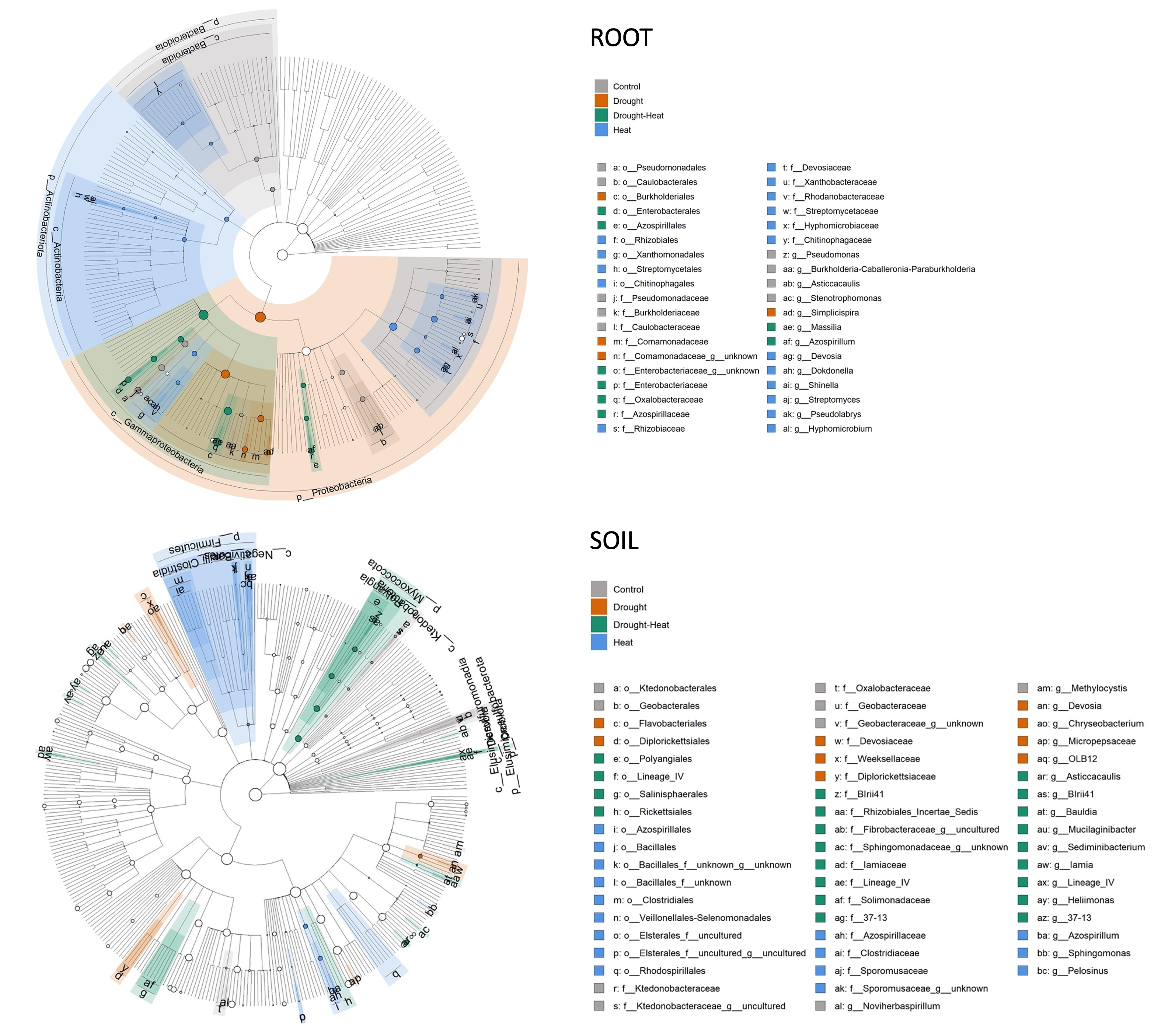

Supplement: Supplementary file 7 [file Image2.png]

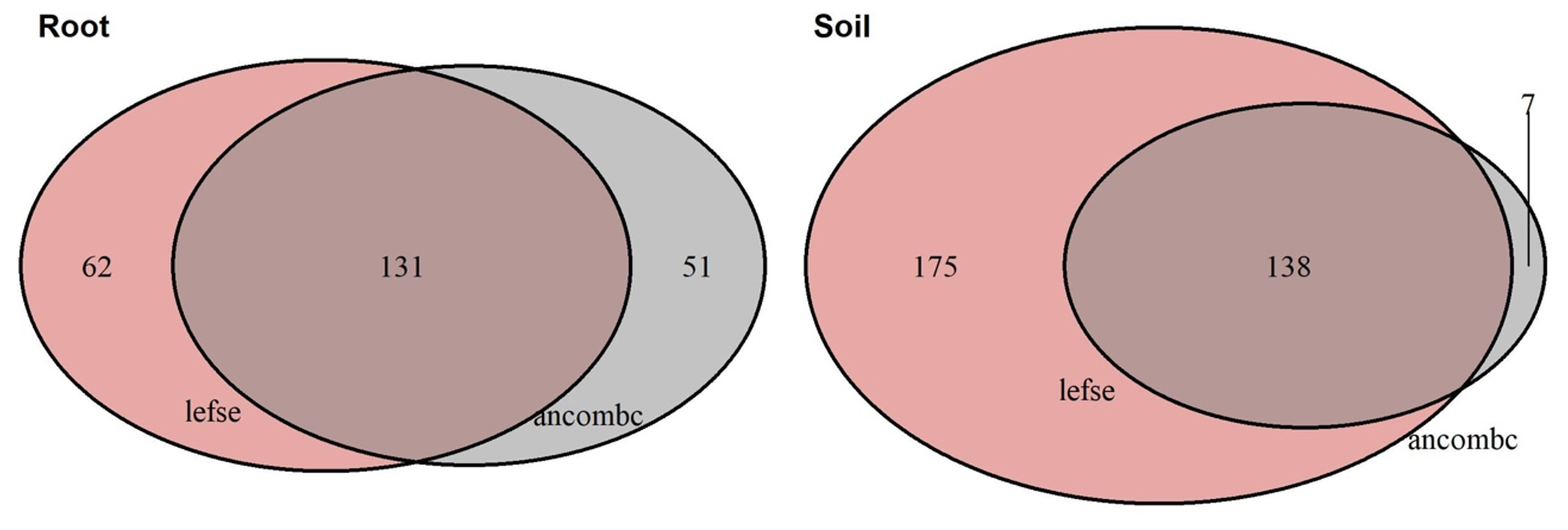

Supplement: Supplementary file 8 [file Image3.png]

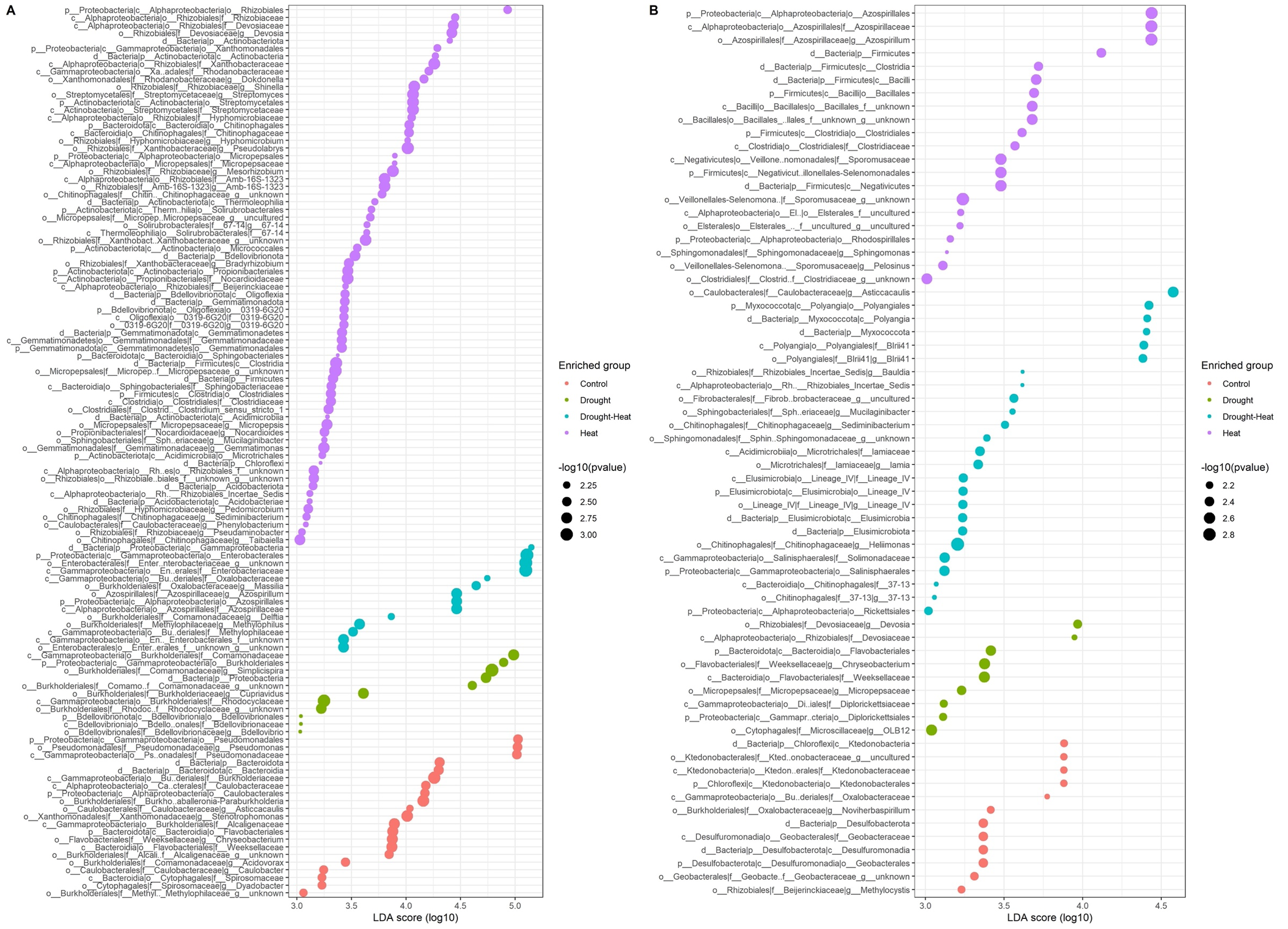

Supplement: Supplementary file 9 [file Image4.png]

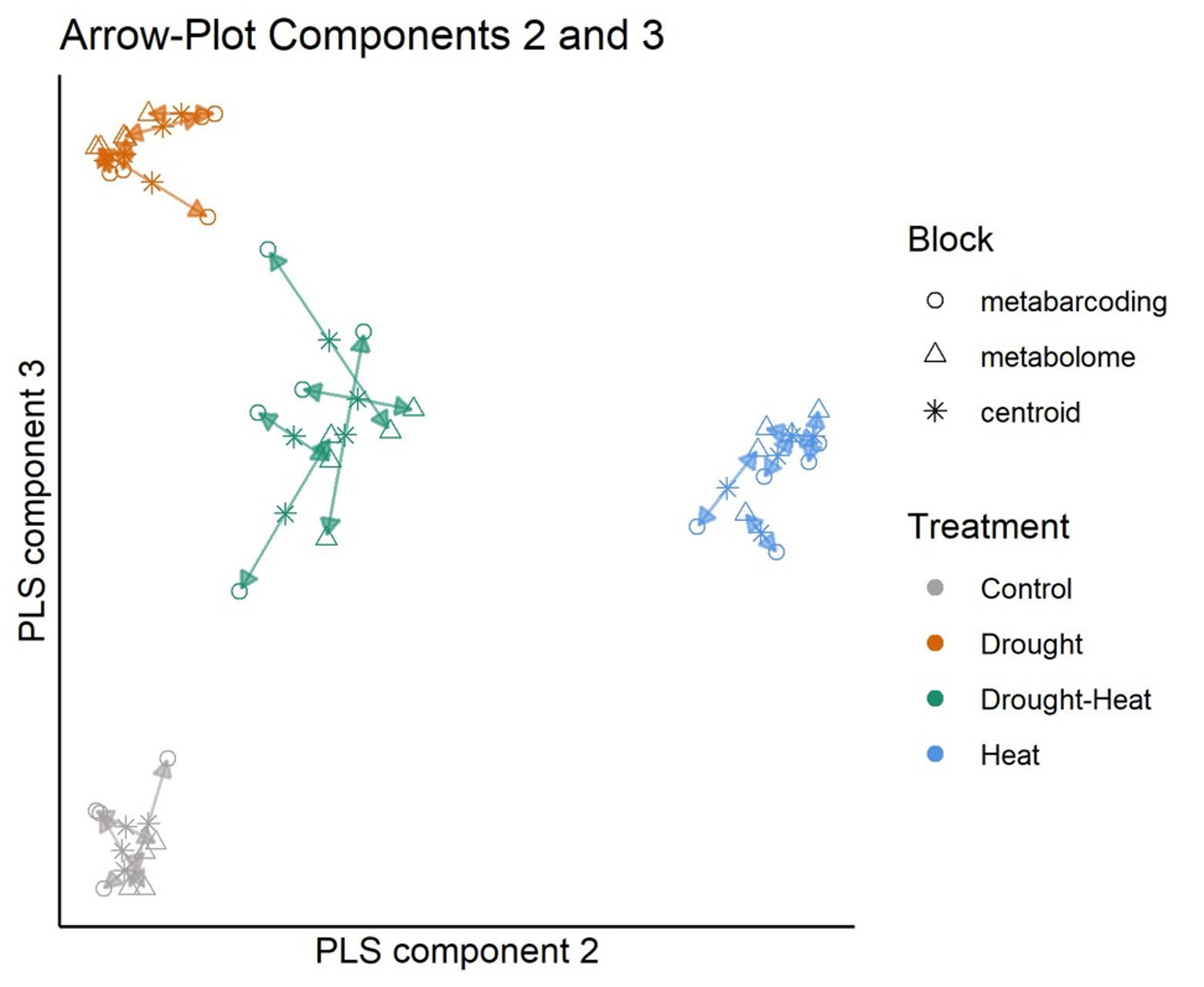

Supplement: Supplementary file 10 [file Image5.png]
